# State Medicaid Coverage for Tobacco Cessation Treatments and Barriers to Accessing Treatments — United States, 2018–2022

**DOI:** 10.15585/mmwr.mm7314a2

**Published:** 2024-04-11

**Authors:** Anne DiGiulio, Michael A. Tynan, Anna Schecter, Kisha-Ann S. Williams, Brenna VanFrank

**Affiliations:** ^1^American Lung Association, Chicago, Illinois; ^2^Office on Smoking and Health, National Center for Chronic Disease Prevention and Health Promotion, CDC.

**Summary**

**What is already known about this topic?**

More than one in five adults enrolled in Medicaid smokes cigarettes. Comprehensive, barrier-free insurance coverage of tobacco cessation treatments can increase smoking cessation.

**What is added by this report?**

From 2018 to 2022, the number of states with comprehensive Medicaid coverage of tobacco cessation treatment increased from 15 to 20; states with no treatment access barriers increased from two to three. Coverage gaps and access barriers remain in many states.

**What are the implications for public health practice?**

State Medicaid programs can improve the health of enrollees who smoke and potentially reduce health care expenditures by providing barrier-free coverage of all evidence-based tobacco cessation treatments and promoting this coverage to enrollees and providers.

## Abstract

The prevalence of cigarette smoking among U.S. adults enrolled in Medicaid is higher than among adults with private insurance; more than one in five adults enrolled in Medicaid smokes cigarettes. Smoking cessation reduces the risk for smoking-related disease and death. Effective treatments for smoking cessation are available, and comprehensive, barrier-free insurance coverage of these treatments can increase cessation. However, Medicaid treatment coverage and treatment access barriers vary by state. The American Lung Association collected and analyzed state-level information regarding coverage for nine tobacco cessation treatments and seven access barriers for standard Medicaid enrollees. As of December 31, 2022, a total of 20 state Medicaid programs provided comprehensive coverage (all nine treatments), an increase from 15 as of December 31, 2018. Only three states had zero access barriers, an increase from two; all three also had comprehensive coverage. Although states continue to improve smoking cessation treatment coverage and decrease access barriers for standard Medicaid enrollees, coverage gaps and access barriers remain in many states. State Medicaid programs can improve the health of enrollees who smoke and potentially reduce health care expenditures by providing barrier-free coverage of all evidence-based cessation treatments and by promoting this coverage to enrollees and providers.

## Introduction

Although the prevalence of cigarette smoking among U.S. adults has been declining for decades (reaching 11.5% in 2021), tobacco-related disparities persist among population groups (*1*). In 2021, smoking prevalence among adults enrolled in Medicaid (21.5%) was higher than it was among adults with private insurance (8.6%) (*1*). In addition, although interest in quitting and quit attempts are similar among adults enrolled in Medicaid and those with private insurance, successful cessation prevalence is lower among those enrolled in Medicaid (*2*). The high prevalence of smoking in this population not only contributes to a substantial health burden for this population but also to the cost of health care. Smoking-attributable health care spending was $225 billion in 2014, more than one half of which was paid by Medicare and Medicaid (*3*).

Effective treatments for smoking cessation include seven Food and Drug Administration (FDA)–approved medications* as well as individual, group, and telephone counseling (*4*). The U.S. Surgeon General has concluded that “insurance coverage for smoking cessation treatment that is comprehensive, barrier-free, and widely promoted increases the use of these treatment services, leads to higher rates of successful quitting, and is cost-effective” (*4*). Although states are required to provide Medicaid expansion^†^ enrollees with coverage for all tobacco cessation treatments,^§^ coverage for standard (i.e., traditional) Medicaid enrollees varies. Standard Medicaid enrollees are persons enrolled in Medicaid under traditional Medicaid eligibility criteria (e.g., low-income pregnant women, children, and persons with a disability), as opposed to Group XIII, or expansion, eligibility. Nationwide, approximately 80% of Medicaid enrollees are covered under standard Medicaid.^¶^ To assess cessation coverage policies among Medicaid programs, the American Lung Association collects state-level** information regarding coverage for nine tobacco cessation treatments^††^ and seven access barriers^§§^ for standard Medicaid enrollees.

## Methods

During January 1, 2019, to December 31, 2022, the American Lung Association compiled data regarding state Medicaid tobacco cessation coverage from state Medicaid websites, Medicaid managed care plan member websites, provider websites, handbooks, policy manuals, plan formularies, preferred drug lists, Medicaid state plan amendments, regulations, and laws.^¶¶^ Analysts contacted personnel from state Medicaid agencies, state health departments, or other state government agencies to verify the information collected, retrieve missing documents, and reconcile discrepancies. Information provided by state personnel was considered accurate. As previously published, comprehensive coverage was defined as coverage of all nine assessed treatments (*5*). Barrier-free coverage was defined as having none of the seven assessed treatment access barriers. Summary statistics were generated and compared with data previously reported through December 31, 2018 (*5*). This activity was reviewed by CDC, deemed research not involving human subjects, and was conducted consistent with applicable federal law and CDC policy.***

## Results

### Coverage of Tobacco Cessation Treatment

As of December 31, 2022, all 50 states and the District of Columbia (DC) covered at least one cessation treatment for all standard Medicaid enrollees, which had not changed since December 31, 2018. As of December 2022, a total of 21 states covered both individual and group counseling for all standard Medicaid enrollees, an increase from 16 states in December 2018 (Table 1). Forty-three states covered all seven medications as of December 2022, an increase from 36 in December 2018 (Table 2). Two states (Delaware and Utah), which had covered all seven medications for all standard enrollees in 2018, no longer did so as of 2022 (four medications in Delaware and two medications in Utah changed from being covered for all standard enrollees to being covered for only some standard enrollees). All 15 states that had provided comprehensive coverage as of December 2018 maintained that coverage through December 2022. Five states (Illinois, New York, North Dakota, Pennsylvania, and Virginia) added comprehensive coverage during the study period.

**TABLE 1:** Coverage of tobacco cessation counseling for standard Medicaid enrollees,* by state^†^ — United States, 2018^§^ and 2022^¶^

State	Coverage and year			
	Individual counseling		Group counseling	
	2018	2022	2018	2022
Alabama	P	P	No	No
Alaska	Yes	Yes	No	No
Arizona	P	V	No	V
Arkansas	Yes	Yes	No	No
California	Yes	Yes	Yes	Yes
Colorado	Yes	Yes	Yes	Yes
Connecticut	Yes	Yes	Yes	Yes
Delaware	Yes	Yes	No	Yes
District of Columbia	Yes	Yes	No	No
Florida	V	Yes	V	V
Georgia	Yes	V	V	V
Hawaii	Yes	Yes	V	V
Idaho	Yes	Yes	No	No
Illinois	V	Yes	No	Yes
Indiana	Yes	Yes	Yes	Yes
Iowa	V	V	V	No
Kansas	Yes	Yes	Yes	Yes
Kentucky	Yes	Yes	Yes	Yes
Louisiana	Yes	P	V	V
Maine	Yes	Yes	Yes	Yes
Maryland	Yes	V	No	V
Massachusetts	Yes	Yes	Yes	Yes
Michigan	Yes	Yes	V	No
Minnesota	Yes	Yes	Yes	Yes
Mississippi	P	P	V	No
Missouri	Yes	Yes	Yes	Yes
Montana	Yes	Yes	No	No
Nebraska	Yes	Yes	V	No
Nevada	V	Yes	V	No
New Hampshire	Yes	V	V	No
New Jersey	V	V	V	V
New Mexico	V	V	V	V
New York	Yes	Yes	Yes	Yes
North Carolina	Yes	Yes	No	V
North Dakota	P	Yes	No	Yes
Ohio	Yes	Yes	Yes	Yes
Oklahoma	Yes	Yes	No	No
Oregon	Yes	Yes	Yes	Yes
Pennsylvania	Yes	Yes	V	Yes
Rhode Island	Yes	Yes	Yes	Yes
South Carolina	Yes	Yes	Yes	Yes
South Dakota	P	Yes	No	No
Tennessee	V	V	No	V
Texas	V	Yes	V	V
Utah	Yes	Yes	P	V
Vermont	Yes	Yes	No	No
Virginia	V	Yes	V	Yes
Washington	V	P	No	No
West Virginia	Yes	Yes	V	No
Wisconsin	Yes	Yes	Yes	Yes
Wyoming	Yes	Yes	No	No
**Totals**				
**Yes**	**36**	**39**	**16**	**21**
**No**	**0**	**0**	**18**	**18**
**V**	**10**	**8**	**16**	**12**
**P**	**5**	**4**	**1**	**0**

**Abbreviations:** P = pregnant; V = varied coverage.

* “Yes” indicates treatment is covered for all standard Medicaid enrollees; “No” indicates treatment is not covered for any standard Medicaid enrollee; “V” indicates treatment coverage varies, with treatment covered for some, but not all, standard Medicaid enrollees; and “P” indicates treatment is covered for pregnant women only.

^†^ Includes the District of Columbia.

^§ ^As of December 31, 2018.

^¶^ As of December 31, 2022.

**TABLE 2:** Coverage of tobacco cessation medications for standard Medicaid enrollees,* by state^†^ — United States, 2018^§^ and 2022^¶^

State	Coverage and year													
	Nicotine patch		Nicotine gum		Nicotine lozenge		Nicotine nasal spray		Nicotine oral inhaler		Bupropion		Varenicline	
	2018	2022	2018	2022	2018	2022	2018	2022	2018	2022	2018	2022	2018	2022
Alabama	Yes	Yes	Yes	Yes	Yes	Yes	Yes	Yes	Yes	Yes	Yes	Yes	Yes	Yes
Alaska	Yes	Yes	Yes	Yes	Yes	Yes	Yes	Yes	Yes	Yes	Yes	Yes	Yes	Yes
Arizona	Yes	Yes	Yes	Yes	Yes	Yes	Yes	Yes	Yes	Yes	Yes	Yes	Yes	Yes
Arkansas	Yes	Yes	Yes	Yes	No	Yes	No	Yes	No	Yes	Yes	Yes	Yes	Yes
California	Yes	Yes	Yes	Yes	Yes	Yes	Yes	Yes	Yes	Yes	Yes	Yes	Yes	Yes
Colorado	Yes	Yes	Yes	Yes	Yes	Yes	Yes	Yes	Yes	Yes	Yes	Yes	Yes	Yes
Connecticut	Yes	Yes	Yes	Yes	Yes	Yes	Yes	Yes	Yes	Yes	Yes	Yes	Yes	Yes
Delaware	Yes	Yes	Yes	Yes	Yes	Yes	Yes	V	Yes	V	Yes	V	Yes	V
District of Columbia	Yes	Yes	Yes	Yes	Yes	Yes	V	V	V	V	Yes	Yes	V	Yes
Florida	Yes	Yes	Yes	Yes	Yes	Yes	No	No	No	No	Yes	Yes	Yes	Yes
Georgia	Yes	Yes	Yes	Yes	Yes	Yes	V	V	V	V	Yes	V	V	V
Hawaii	Yes	Yes	Yes	Yes	V	V	V	V	V	V	Yes	Yes	Yes	Yes
Idaho	Yes	Yes	Yes	Yes	Yes	Yes	Yes	Yes	Yes	Yes	Yes	Yes	Yes	Yes
Illinois	Yes	Yes	Yes	Yes	Yes	Yes	V	Yes	V	Yes	Yes	Yes	V	Yes
Indiana	Yes	Yes	Yes	Yes	Yes	Yes	Yes	Yes	Yes	Yes	Yes	Yes	Yes	Yes
Iowa	Yes	Yes	Yes	Yes	Yes	Yes	Yes	Yes	Yes	Yes	Yes	Yes	Yes	Yes
Kansas	Yes	Yes	Yes	Yes	Yes	Yes	Yes	Yes	Yes	Yes	Yes	Yes	Yes	Yes
Kentucky	Yes	Yes	Yes	Yes	Yes	Yes	Yes	Yes	Yes	Yes	Yes	Yes	Yes	Yes
Louisiana	Yes	Yes	Yes	Yes	Yes	Yes	V	Yes	V	Yes	Yes	Yes	V	Yes
Maine	Yes	Yes	Yes	Yes	Yes	Yes	Yes	Yes	Yes	Yes	Yes	Yes	Yes	Yes
Maryland	Yes	Yes	Yes	Yes	Yes	Yes	Yes	Yes	Yes	Yes	Yes	Yes	Yes	Yes
Massachusetts	Yes	Yes	Yes	Yes	Yes	Yes	Yes	Yes	Yes	Yes	Yes	Yes	Yes	Yes
Michigan	Yes	Yes	Yes	Yes	Yes	Yes	Yes	Yes	Yes	Yes	Yes	Yes	Yes	Yes
Minnesota	Yes	Yes	Yes	Yes	Yes	Yes	Yes	Yes	Yes	Yes	Yes	Yes	Yes	Yes
Mississippi	Yes	Yes	Yes	Yes	Yes	Yes	Yes	Yes	Yes	Yes	Yes	Yes	Yes	Yes
Missouri	Yes	Yes	Yes	Yes	Yes	Yes	Yes	Yes	Yes	Yes	Yes	Yes	Yes	Yes
Montana	Yes	Yes	Yes	Yes	Yes	Yes	Yes	Yes	Yes	Yes	Yes	Yes	Yes	Yes
Nebraska	Yes	Yes	Yes	Yes	Yes	Yes	Yes	Yes	Yes	Yes	Yes	Yes	Yes	Yes
Nevada	Yes	Yes	Yes	Yes	Yes	Yes	V	V	Yes	V	Yes	Yes	Yes	V
New Hampshire	Yes	Yes	Yes	Yes	Yes	Yes	Yes	Yes	Yes	Yes	Yes	Yes	Yes	Yes
New Jersey	Yes	Yes	Yes	Yes	Yes	Yes	V	Yes	V	Yes	Yes	Yes	Yes	Yes
New Mexico	Yes	Yes	Yes	Yes	Yes	Yes	V	Yes	V	Yes	Yes	Yes	Yes	Yes
New York	Yes	Yes	Yes	Yes	Yes	Yes	Yes	Yes	V	Yes	Yes	Yes	Yes	Yes
North Carolina	Yes	Yes	Yes	Yes	Yes	Yes	Yes	Yes	Yes	Yes	Yes	Yes	Yes	Yes
North Dakota	Yes	Yes	Yes	Yes	Yes	Yes	Yes	Yes	Yes	Yes	Yes	Yes	Yes	Yes
Ohio	Yes	Yes	Yes	Yes	Yes	Yes	Yes	Yes	Yes	Yes	Yes	Yes	Yes	Yes
Oklahoma	Yes	Yes	Yes	Yes	Yes	Yes	Yes	Yes	Yes	Yes	Yes	Yes	Yes	Yes
Oregon	Yes	Yes	Yes	Yes	Yes	Yes	Yes	Yes	Yes	Yes	Yes	Yes	Yes	Yes
Pennsylvania	Yes	Yes	Yes	Yes	Yes	Yes	V	Yes	V	Yes	Yes	Yes	Yes	Yes
Rhode Island	Yes	Yes	Yes	Yes	Yes	Yes	Yes	Yes	Yes	Yes	Yes	Yes	Yes	Yes
South Carolina	Yes	Yes	Yes	Yes	Yes	Yes	Yes	Yes	Yes	Yes	Yes	Yes	Yes	Yes
South Dakota	No	No	No	No	No	No	No	No	No	No	Yes	Yes	Yes	Yes
Tennessee	Yes	Yes	Yes	Yes	Yes	Yes	Yes	Yes	Yes	Yes	Yes	Yes	Yes	Yes
Texas	Yes	Yes	Yes	Yes	Yes	Yes	Yes	Yes	Yes	Yes	Yes	Yes	Yes	Yes
Utah	Yes	Yes	Yes	Yes	Yes	Yes	Yes	V	Yes	V	Yes	Yes	Yes	Yes
Vermont	Yes	Yes	Yes	Yes	Yes	Yes	Yes	Yes	Yes	Yes	Yes	Yes	Yes	Yes
Virginia	Yes	Yes	Yes	Yes	V	Yes	V	Yes	V	Yes	Yes	Yes	Yes	Yes
Washington	Yes	Yes	Yes	Yes	Yes	Yes	V	Yes	V	Yes	Yes	Yes	V	Yes
West Virginia	Yes	Yes	Yes	Yes	Yes	Yes	Yes	Yes	Yes	Yes	Yes	Yes	Yes	Yes
Wisconsin	Yes	Yes	Yes	Yes	Yes	Yes	Yes	Yes	Yes	Yes	Yes	Yes	Yes	Yes
Wyoming	Yes	Yes	Yes	Yes	Yes	Yes	Yes	Yes	Yes	Yes	Yes	Yes	Yes	Yes
**Totals**														
**Yes**	**50**	**50**	**50**	**50**	**47**	**49**	**37**	**43**	**37**	**43**	**51**	**49**	**46**	**48**
**No**	**1**	**1**	**1**	**1**	**2**	**1**	**3**	**2**	**3**	**2**	**0**	**0**	**0**	**0**
**V**	**0**	**0**	**0**	**0**	**2**	**1**	**11**	**6**	**11**	**6**	**0**	**2**	**5**	**3**

**Abbreviation:** V = varied coverage.

* “Yes” indicates treatment is covered for all standard Medicaid enrollees; “No” indicates treatment is not covered for any standard Medicaid enrollee; and “V” indicates treatment coverage varies, with treatment covered for some, but not all, standard Medicaid enrollees.

^†^ Includes the District of Columbia.

^§ ^As of December 31, 2018.

^¶^ As of December 31, 2022.

### Treatment Access Barriers

During December 2018–December 2022, the number of states with a treatment access barrier decreased for all seven barriers. For example, the number of states not requiring copayments increased from 28 to 39. However, some barriers continue to be common. As of December 2022, the three most common barriers (that apply to all or some standard Medicaid enrollees) were duration limits (39 states; 76%), annual limits on the number of covered quit attempts (35; 69%), and requirement for prior authorization (30; 59%) (Table 3). These three barriers were also the most common in December 2018. As of December 2022, only three states (Kentucky, Missouri, and Wisconsin) provided barrier-free coverage, an increase from two (Kentucky and Missouri) in December 2018. All three of these states provided comprehensive coverage.

**TABLE 3:** Barriers* to coverage for tobacco cessation treatments for standard Medicaid enrollees,^†^ by state^§^ — United States, 2018^¶^ and 2022**

State	Coverage barrier and year													
	Copayments required		Prior authorization required		Counseling required for medications		Stepped care therapy		Limits on duration		Annual limit on quit attempts		Lifetime limit on quit attempts	
	2018	2022	2018	2022	2018	2022	2018	2022	2018	2022	2018	2022	2018	2022
Alabama	No	Yes	Yes	Yes	No	Yes	No	No	Yes	Yes	Yes	Yes	No	No
Alaska	Yes	Yes	No	Yes	No	No	No	No	Yes	Yes	Yes	Yes	No	No
Arizona	No	V	No	No	No	V	No	No	Yes	Yes	Yes	V	No	No
Arkansas	No	No	Yes	Yes	Yes	Yes	No	No	V	No	Yes	No	No	No
California	No	No	V	V	No	No	No	No	V	V	V	V	No	No
Colorado	No	No	No	No	Yes	No	No	No	Yes	Yes	Yes	Yes	No	No
Connecticut	No	No	Yes	Yes	No	No	No	No	Yes	Yes	No	Yes	No	No
Delaware	No	No	V	V	V	V	V	V	V	V	V	V	No	No
District of Columbia	V	No	V	V	No	No	No	No	V	No	V	No	No	No
Florida	V	No	No	Yes	No	No	No	No	Yes	Yes	Yes	Yes	No	No
Georgia	V	Yes	V	V	No	V	No	V	Yes	V	Yes	V	No	No
Hawaii	No	No	V	V	Yes	V	V	No	V	Yes	Yes	Yes	No	No
Idaho	No	No	Yes	Yes	Yes	No	Yes	Yes	Yes	No	Yes	No	No	No
Illinois	V	No	V	No	No	No	V	No	V	V	V	V	No	No
Indiana	Yes	No	V	No	Yes	Yes	V	V	Yes	V	Yes	V	No	No
Iowa	No	No	Yes	No	Yes	No	Yes	No	Yes	Yes	Yes	Yes	No	No
Kansas	No	No	No	No	No	No	No	No	Yes	Yes	Yes	Yes	No	No
Kentucky	No	No	No	No	No	No	No	No	No	No	No	No	No	No
Louisiana	V	No	V	Yes	No	Yes	No	Yes	V	Yes	V	Yes	No	No
Maine	No	No	Yes	Yes	No	No	Yes	Yes	No	No	No	No	No	No
Maryland	No	No	Yes	Yes	No	No	Yes	Yes	Yes	Yes	No	Yes	No	No
Massachusetts	Yes	No	Yes	No	No	No	No	No	Yes	Yes	Yes	Yes	No	No
Michigan	No	No	No	V	No	No	No	No	V	V	No	V	No	No
Minnesota	No	No	V	Yes	No	No	No	No	V	No	No	No	No	No
Mississippi	V	V	Yes	No	No	No	No	No	No	V	Yes	No	No	No
Missouri	No	No	No	No	No	No	No	No	No	No	No	No	No	No
Montana	No	No	Yes	Yes	No	No	Yes	No	Yes	Yes	Yes	No	No	No
Nebraska	V	V	Yes	V	Yes	No	No	No	Yes	Yes	Yes	Yes	No	No
Nevada	No	No	V	V	No	No	No	No	Yes	V	V	No	No	No
New Hampshire	V	No	V	V	V	No	V	V	V	V	V	V	No	No
New Jersey	V	No	No	No	No	No	No	No	V	No	V	NA	No	No
New Mexico	V	No	V	No	V	No	No	No	V	V	Yes	V	No	No
New York	V	V	No	V	No	No	No	No	No	No	No	No	No	No
North Carolina	Yes	Yes	No	No	No	No	Yes	Yes	Yes	Yes	No	Yes	No	No
North Dakota	Yes	No	Yes	No	Yes	No	Yes	Yes	Yes	Yes	Yes	Yes	No	No
Ohio	No	No	V	No	No	V	V	No	V	V	V	Yes	No	No
Oklahoma	No	No	No	No	No	No	No	No	Yes	Yes	No	Yes	No	No
Oregon	No	No	Yes	V	No	No	No	V	Yes	Yes	Yes	V	No	No
Pennsylvania	V	No	V	Yes	No	No	V	No	V	Yes	V	Yes	No	No
Rhode Island	No	No	V	V	V	No	No	V	V	V	No	V	No	No
South Carolina	No	No	No	No	No	No	No	No	Yes	Yes	Yes	Yes	No	No
South Dakota	Yes	Yes	No	No	No	No	No	No	No	Yes	No	No	No	No
Tennessee	Yes	Yes	Yes	Yes	No	No	Yes	Yes	Yes	Yes	Yes	Yes	V	No
Texas	No	No	Yes	Yes	No	No	Yes	Yes	V	No	V	V	No	No
Utah	Yes	V	V	V	V	V	V	V	V	V	Yes	V	No	No
Vermont	No	No	Yes	Yes	No	No	Yes	Yes	Yes	Yes	Yes	No	No	No
Virginia	V	No	V	No	No	No	V	No	No	Yes	No	No	No	No
Washington	No	No	V	Yes	V	No	V	No	V	No	V	Yes	V	No
West Virginia	No	No	Yes	Yes	Yes	Yes	Yes	Yes	Yes	Yes	Yes	Yes	No	V
Wisconsin	Yes	No	No	No	No	No	No	No	Yes	No	No	No	No	No
Wyoming	Yes	V	No	No	No	No	No	No	Yes	Yes	Yes	Yes	No	No
**Totals**														
**Yes**	**10**	**6**	**17**	**17**	**9**	**5**	**11**	**10**	**26**	**26**	**25**	**22**	**0**	**0**
**No**	**28**	**39**	**16**	**21**	**36**	**40**	**30**	**34**	**7**	**12**	**14**	**15**	**49**	**50**
**V**	**13**	**6**	**18**	**13**	**6**	**6**	**10**	**7**	**18**	**13**	**12**	**13**	**2**	**1**
**NA**	**0**	**0**	**0**	**0**	**0**	**0**	**0**	**0**	**0**	**0**	**0**	**1**	**0**	**0**

**Abbreviations:** NA = not available; V = varied coverage.

* Barriers apply to one or more cessation treatments.

^†^ “Yes” indicates a barrier applies to all standard Medicaid enrollees; “No” indicates a barrier does not apply to any standard Medicaid enrollee; and “V” indicates a barrier applies to some, but not all, standard Medicaid enrollees.

^§^ Includes the District of Columbia.

^¶ ^As of December 31, 2018.

** As of December 31, 2022.

## Discussion

During 2018–2022, states continued to add coverage of tobacco cessation treatments and to remove treatment access barriers for standard Medicaid enrollees. However, coverage gaps and access barriers remain in many states. Although the number of states with comprehensive coverage increased from 15 in 2018 to 20 in 2022, this increase falls short of the Healthy People 2030 target of all 50 states and DC.^†††^ In 2022, only three states provided coverage without any barriers. Increasing cessation coverage and decreasing barriers increases access to effective treatments that can increase the likelihood of successful quitting and improve health outcomes for persons who smoke (*4*).

The increase in the number of states with comprehensive treatment coverage and without barriers is likely related to state legislative actions. For example, Ohio passed legislation in 2020 requiring the state Medicaid program to cover a comprehensive cessation benefit with minimal barriers; Illinois passed similar legislation in 2021.^§§§^ These laws not only improve coverage and removed barriers, but also ensure that managed care plans will maintain this level of coverage in the future, even if new carriers are selected via competitive state bidding processes.

Laws like those passed in Ohio and Illinois can also help standardize tobacco cessation benefits across plans within a state. In the absence of such laws, treatment coverage and barriers can vary within a state’s Medicaid program, potentially limiting treatment access. Different Medicaid-managed care plans within a state can set different coverage policies. Consistent comprehensive coverage of tobacco cessation treatments with minimal barriers has the potential to increase standard Medicaid enrollees’ access to treatments and minimize confusion for both enrollees and providers.

Improved cessation treatment coverage observed in this study might also be related to some states^¶¶¶^ implementing Medicaid expansion during the study period (*6*). Many state Medicaid programs provide the same coverage for standard and expansion enrollees (*7*). Since states are required to provide expansion enrollees with coverage of all cessation treatments, consistency of coverage between standard and expansion plans might result in improvements in coverage for standard enrollees. Medicaid expansion has been shown to support cessation; states that have implemented Medicaid expansion have witnessed an increase in smoking cessation among lower-income adults (*8*,*9*). Opportunities remain for all states to improve coverage and increase promotion of available tobacco cessation benefits to encourage and support successful quitting.

This study demonstrates continued progress in decreasing tobacco cessation treatment access barriers for standard Medicaid enrollees. The biggest improvement in barrier removal was for copayments, with a nearly one third increase in the number of states without copayment requirements. One potential contributor to this change was enactment of the Families First Coronavirus Response Act (FFCRA),**** which increased the federal share of Medicaid spending by 6.2% with the requirement that states limit new cost-sharing for Medicaid enrollees. Continued monitoring of treatment access barriers remains important, particularly because the FFCRA maintenance of effort requirement, which limited cost-sharing, ended in 2023.^††††^ How this change in policy might affect access barriers for cessation treatments is unknown.

### Limitations

The findings in this report are subject to at least two limitations. First, Medicaid-managed care plans can change with little notice and can vary widely between plans, which can make determining up-to-date coverage challenging. Second, information provided by state personnel could not be verified, potentially resulting in data misclassification.

### Implications for Public Health Practice

More than one in five adults enrolled in Medicaid smoke cigarettes (*1*). Increasing comprehensive, barrier-free tobacco cessation insurance coverage for the more than 48 million adults enrolled in Medicaid^§§§§^ has the potential to reduce tobacco-related disparities in this population by increasing access to and usage of treatments that help persons quit smoking (*4*). By providing barrier-free coverage of all evidence-based tobacco cessation treatments, and promoting this coverage to enrollees and providers, state Medicaid programs can improve the health of enrollees who smoke and potentially reduce health care expenditures.

## Footnotes

* These include five nicotine replacement therapies (nicotine patch, gum, lozenge, nasal spray, and oral inhaler) and two non-nicotine medications (bupropion and varenicline).

† Medicaid expansion, also known as Group XIII eligibility, provides Medicaid coverage to persons ineligible for standard Medicaid who have an income ≤138% of the federal poverty level. Medicaid expansion was created by the Patient Protection and Affordable Care Act and implemented in 2014. https://www.healthcare.gov/medicaid-chip/getting-medicaid-chip/

§ The Patient Protection and Affordable Care Act (ACA) requires Medicaid expansion plans to cover treatment given an “A” or “B” grade by the U.S. Preventive Services Task Force without cost-sharing (https://www.congress.gov/111/plaws/publ148/PLAW-111publ148.pdf). Tobacco cessation currently receives an “A” grade (https://www.uspreventiveservicestaskforce.org/uspstf/recommendation/tobacco-use-in-adults-and-pregnant-women-counseling-and-interventions) and is included in the ACA requirement (https://www.cms.gov/cciio/resources/fact-sheets-and-faqs/aca_implementation_faqs19). Currently, this requirement is being legally challenged. https://www.kff.org/womens-health-policy/issue-brief/explaining-litigation-challenging-the-acas-preventive-services-requirements-braidwood-management-inc-v-becerra/

¶
https://www.kff.org/medicaid/issue-brief/medicaid-expansion-enrollment-and-spending-leading-up-to-the-covid-19-pandemic/

** The term “states” includes DC.

†† Treatments include seven FDA-approved smoking cessation medications and two types of counseling (individual and group). Telephone counseling was not examined because it is available free to callers (including Medicaid enrollees) via state quitlines in all 50 states and DC.

§§ Barriers to treatment include requirements for copayment, prior authorization, counseling for medications, and stepped care therapy, and limits on the duration and number (both annual and lifetime) of covered quit attempts. A barrier was considered to be in place if it existed for any of the nine assessed cessation treatments.

¶¶ Information on state Medicaid cessation coverage compiled by the American Lung Association is available in the CDC State Activities Tracking and Evaluation (STATE) System. Some data presented in this report differ from data available in the STATE System because of differences in coding rules, categories, and reporting periods. https://www.cdc.gov/statesystem

*** 45 C.F.R. part 46; 21 C.F.R. part 56; 42 U.S.C. Sect. 241(d), 5 U.S.C. Sect. 552a, 44 U.S.C. Sect. 3501 et seq.

†††
https://health.gov/healthypeople/objectives-and-data/browse-objectives/tobacco-use/increase-medicaid-coverage-evidence-based-treatment-help-people-quit-using-tobacco-tu-16

§§§ https://www.legislature.ohio.gov/legislation/133/hb11; https://www.ilga.gov/legislation/BillStatus.asp?DocNum=2294&GAID=16&DocTypeID=SB&SessionID=110&GA=102

¶¶¶ During the study period, Medicaid expansion occurred in Maine and Virginia (2019); Idaho, Nebraska, and Utah (2020); and Missouri and Oklahoma (2021).

**** The Centers for Medicare & Medicaid Services has issued guidance to states on implementing this provision (https://www.medicaid.gov/state-resource-center/downloads/covid-19-faqs.pdf). The FFCRA included a maintenance of effort requirement, meaning that states could not disenroll persons from Medicaid or impose new cost-sharing for Medicaid enrollees while the federal Medicaid payment was increased by 6.2%. www.congress.gov/116/plaws/publ127/PLAW-116publ127.pdf

††††
https://www.medicaid.gov/federal-policy-guidance/downloads/sho23002.pdf

§§§§ Includes both standard and expansion Medicaid enrollees. https://www.medicaid.gov/sites/default/files/2023-03/December-2022-medicaid-chip-enrollment-trend-snapshot.pdf
